# A Comprehensive Review: Sphingolipid Metabolism and Implications of Disruption in Sphingolipid Homeostasis

**DOI:** 10.3390/ijms22115793

**Published:** 2021-05-28

**Authors:** Brianna M. Quinville, Natalie M. Deschenes, Alex E. Ryckman, Jagdeep S. Walia

**Affiliations:** Centre for Neuroscience Studies at Queen’s University, Kingston, ON K7L 3N6, Canada; 12nmd4@queensu.ca (N.M.D.); 14aer1@queensu.ca (A.E.R.)

**Keywords:** sphingolipid, sphingosine-1-phosphate, ceramide, glycosphingolipids, neurodegeneration, inflammation, lysosomal storage disorder, biosynthesis, glycosyl hydrolase

## Abstract

Sphingolipids are a specialized group of lipids essential to the composition of the plasma membrane of many cell types; however, they are primarily localized within the nervous system. The amphipathic properties of sphingolipids enable their participation in a variety of intricate metabolic pathways. Sphingoid bases are the building blocks for all sphingolipid derivatives, comprising a complex class of lipids. The biosynthesis and catabolism of these lipids play an integral role in small- and large-scale body functions, including participation in membrane domains and signalling; cell proliferation, death, migration, and invasiveness; inflammation; and central nervous system development. Recently, sphingolipids have become the focus of several fields of research in the medical and biological sciences, as these bioactive lipids have been identified as potent signalling and messenger molecules. Sphingolipids are now being exploited as therapeutic targets for several pathologies. Here we present a comprehensive review of the structure and metabolism of sphingolipids and their many functional roles within the cell. In addition, we highlight the role of sphingolipids in several pathologies, including inflammatory disease, cystic fibrosis, cancer, Alzheimer’s and Parkinson’s disease, and lysosomal storage disorders.

## 1. Introduction

Sphingolipids are a set of structurally diverse lipids originally named after the sphinx in the 1870s, due to their enigmatic structure [1]. Holding both hydrophobic and hydrophilic properties, sphingolipids are important residents of the plasma membrane of almost all vertebrate cells and contribute to a number of different cellular functions [2]. Specifically, processes such as cell–cell interaction, cell adhesion, cell proliferation and migration, and cell death are in part regulated by sphingolipids [2]. These bioactive molecules participate in highly complex and interconnected pathways integral to cellular life. One such example is the sphingolipid rheostat. This major contributor to sphingolipid homeostasis involves sphingolipid intermediates, ceramide and sphingosine-1-phosphate (S1P), which play opposing roles in cell survival and growth [3]. Ceramide has been shown to promote cell death by activating mitochondrial permeabilization, whereby harmful mitochondrial contents, such as caspases, are released into the cytosol and activate apoptotic pathways [4,5]. S1P is a well-known anti-apoptotic regulator; it has been found to bind the S1P1 receptor and subsequently suppress caspase-3 activity, thus preventing apoptosis [6].

Disruption or dysregulation of sphingolipid homeostasis can result in detrimental pathophysiological conditions. It is now recognized that sphingolipids are involved in inflammatory processes, neurodegeneration, cancer metastasis, and lysosomal storage disorders (LSDs) [7,8,9]. In the current review we will specifically touch on the sphingolipid rheostat’s involvement in cancer and Alzheimer’s and Parkinson’s disease, as well as briefly discussing the role sphingolipids play as a storage substrate in LSDs.

## 2. Sphingolipid Structures

Sphingolipids are a class of amphipathic lipids which share a sphingoid base backbone that is N-acylated with various fatty acid chains. This group includes lipids such as sphingosine, ceramide, S1P, ceramide-1-phosphate (C1P), and sphingomyelin (SM). Sphingolipids can be divided into three structural classes—sphingoid bases and derivatives, ceramides, and complex sphingolipids—in which sphingoid bases act as the structural foundation for all sphingolipid derivatives [2].

### 2.1. Sphingoid Bases and Simple Derivatives

Sphingoid bases, also known as long-chain bases (LCBs), are non-transient amino alcohol precursors to ceramide and complex sphingolipids. In contrast to complex sphingolipid derivatives, the hydrophilic head groups of sphingoid bases consist solely of hydroxyl groups. The most frequently occurring mammalian sphingoid bases include sphingosine (2-amino-4-trans-octadecene-1,3-diol) and dihydrosphingosine ((2*R*,3*S*)-2-aminooctadecane-1,3-diol), commonly known as sphinganine (shown in Table 1). The 18-carbon amino alcohol, sphingosine, is produced through the salvage pathway, following the breakdown of ceramide, whereas sphinganine is synthesized in the *de novo* biosynthesis pathway. These two sphingoid bases differ structurally by a *trans*-double bond located at C4, which is present in sphingosine, but not sphinganine. Variances in sphingoid bases can occur, with the incorporation of alternative amino acid residues that are conditional on specific mutations of serine palmitoyltransferase (SPT), or by the incorporation of variable acyl chain lengths depending on the condensing acyl-CoA (reviewed in [10]).

Phytosphingosine ((2S,3S,4R)-2-aminooctadecane-1,3,4-triol) (PHS) is another mammalian sphingoid base which slightly differs in structure, due to an extra hydroxyl group located at C4, instead of the *trans*-double bond between C4 and C5 seen in sphingosine. Unlike sphingosine and sphinganine, PHS is only found in specific vertebrate tissues such as the epidermis, small intestine, kidney, and epithelial tissues. Although it is relatively rare in animals, it is a bioactive lipid and major backbone of glycosphingolipids in fungi, yeast, and plants [11,12,13,14]. It has been suggested that the extra hydroxyl group found in PHS may give rise to its unique ability to strongly interact with other molecules through an additional hydrogen bond [15]. As a result, PHS permits lipids to stack into lipid lamellae and perform important functions such as reinforcing the permeability barrier found in the stratum corneum of the epidermis. However, the extra hydroxyl group causes PHS metabolism to be quite complex. In fact, the exact metabolic pathway of PHS remains mostly unknown [15].

The sphingoid bases are easily converted to sphingoid base derivatives through small modifications such as phosphorylation and acetylation [16]. Sphingosine, sphinganine, and PHS all possess a terminal hydroxyl group which may be phosphorylated to form sphingosine-1-phosphate [{[(4E)-2-amino-3-hydroxyoctadec-4-en-1-yl]oxy} phosphonic acid] (S1P), sphinganine-1-phosphate [{[(2S,3R)-2-amino-3-hydroxyoctadecyl]oxy}phosphonic acid], and phytosphingosine-1-phosphate [{[(2S,3S,4R)-2-amino-3,4-dihydroxyoctadecyl]oxy}phosphonic acid], respectively.

In addition to the well-known canonical sphingolipids, alternative forms, termed deoxysphingolipids, are produced by mutated forms of SPT. The primary difference between them is the missing hydroxyl group, which is located at C1 in canonical sphingolipids. There are only two identified deoxysphingolipids: 1-deoxysphinganine [[(2S,3R)-3-hydroxyoctadecan-2-yl]azanium] and 1-deoxymethylsphinganine [(2R)-1-aminoheptadecan-2-ol] (shown in Table 1) [17,18]. It is hypothesized that 1-deoxysphinganine could be converted to 1-deoxysphingosine by sphingolipid delta(4)-desaturase 1 (DES1) enzyme [19]. Originally, the structure of 1-deoxysphingosine was assumed to possess a double bond at C4 in the E configuration. Steiner et al. (2016) later confirmed that the native structure of 1-deoxysphingosine has a double bond at C14, with the primary stereoisomer as 14Z (both structures can be seen in Table 1). The synthesis and characterization of 1-deoxysphingosine [(2S,3R,14Z)-2-amino-14-octadecen-3-ol] was further investigated along with 1-deoxymethylsphinganine, which is devoid of the entire hydroxymethylene group [20,21]. In addition, Lone et al. (2020) recently determined the properties and functions of each of the three subunits of SPT—SPTLC1, SPTLC2, and SPTLC3. Interestingly, they discovered a previously undescribed methyl-branched sphingoid base, exclusively produced by the SPTLC3 subunit in humans [22]. Considering that methyl-branched sphingoid bases had thus far only been known to exist in lower-level invertebrates [23], the authors hypothesized that humans may acquire methyl-branched sphingoid bases from their diet or gut microbiota [22].

### 2.2. Ceramide

Ceramides are essential constituents of complex sphingolipids and differ from sphingoid bases in the addition of a long-chain fatty acid to the amine group. Ceramide acts as the backbone of sphingolipids such as SM, cerebrosides, and gangliosides, and as such is an important resident of the plasma membrane of eukaryotic cells. Several ceramide structures exist, influenced by (1) the variable fatty acid chain length and saturation it may possess, ranging from C6 to C26; (2) whether a hydroxyl group or double bond is initially introduced into the sphingoid base; and (3) the length of the sphingoid base, and the possession of a hydroxyl group at the 1-position [24]. As such, it was calculated in 2011 that up to 360 different ceramide structures may exist [25].

Ceramide is inherently hydrophobic due to the hydrocarbon chains it possesses, and thus is insoluble in aqueous solutions, including the cytosol. C18 ceramide is the most abundant ceramide of the nervous system; therefore, in this review, the term ceramide refers henceforth to C18 ceramide specifically. Hydrocarbon chains give rise to ceramide’s ability to stack itself into highly concentrated layers, especially in the human skin barrier, but also within the plasma membrane. As a consequence, ceramide increases the molecular rigidity of the plasma membrane [26,27] and is thought to increase plasma membrane permeability; however, the latter point remains controversial [28]. These properties are also dependent on the acyl chain length of ceramide—small-chain ceramides are more symmetrically shaped and favour a positive curvature in lipid monolayers, whereas long-chain ceramides take on a cone shape and promote a negative curvature of the bilayer, leading to membrane invaginations, which may promote vesiculation or fusion (reviewed in [29]).

### 2.3. Complex Sphingolipids

#### 2.3.1. Phosphosphingolipids

The basic composition of complex sphingolipids includes a ceramide backbone and often a polar head group at position 1. Typically, sphingolipids are categorized based on their head group into two main classes: phosphosphingolipids (PSLs) and glycosphingolipids (GSLs); however, these groups are not mutually exclusive. PSLs can also be considered acidic GSLs, but for the purposes of this review, will only be discussed as their own category.

As the name suggests, PSLs contain the basic sphingolipid structure with the addition of one or more phosphate groups. SM is the most common PSL, made of phosphocholine and ceramide, taking on a cylindrical structure. SM species make up the most prevalent sphingolipids within mammalian cells [30] and are a major component of myelin.

Another PSL is C1P, the primary antagonist to ceramide. Although these structures differ by a single phosphate group, they play opposing functional roles in cells, as discussed in later sections.

#### 2.3.2. Glycosphingolipids

GSLs are structurally composed of a ceramide backbone covalently bound to at least one carbohydrate moiety. In plants, the carbohydrate moiety is frequently a simple sugar such as glucose, whereas in mammals, the carbohydrate can range from a simple sugar to a complex head group, where several carbohydrates or other acidic/neutral moieties may be attached. GSLs encompass a much larger and diverse group of structures and are typically categorized based on charge into neutral and acidic GSLs.

#### 2.3.3. Neutral Glycosphingolipids

Neutral GSLs are composed of three major lipids: glucosylceramide (GlcCer), galactosylceramide (GalCer), and lactosylceramide (LacCer), which mainly serve as simple biosynthetic precursors for more complex derivatives [31]. GlcCer and GalCer—also known as the cerebrosides—are among the most simple GSL structures and consist of a monosaccharide head group attached at the 1-OH position of ceramide [2]. The addition of galactose to GlcCer through a β-1,4-glycosidic linkage produces LacCer, the basis for almost all complex GSLs [2].

#### 2.3.4. Acidic Glycosphingolipids

##### Gangliosides

Acidic GSLs are often considered complex lipids and are accompanied by a negatively charged head group, which determines the structure of the acidic GSL. There are four main categories of acidic GSLs: (1) gangliosides, which possess one or more sialic acid groups; (2) glucuronoglycosphingolipids, which possess a glucuronic acid moiety; (3) sulfatoglycoshpingolipids, which possess a sulfate group; and (4) phosphoglycosphingolipids, which possess a phosphate group [2].

Gangliosides make up an intricate group of lipids which primarily populate the outer leaflet of the plasma membrane of neuronal cells. Their hydrophobic ceramide backbone anchors into the plasma membrane, whereas the oligosaccharide moiety protrudes into the cytosol for molecular interaction. The variability in the carbohydrate-based head group gives rise to over 200 ganglioside structures [32]. Furthermore, the ceramide backbone of a given ganglioside may vary according to the acyl chain length and the degree of saturation, further adding to gangliosides’ structural diversity.

**Figure 1:**
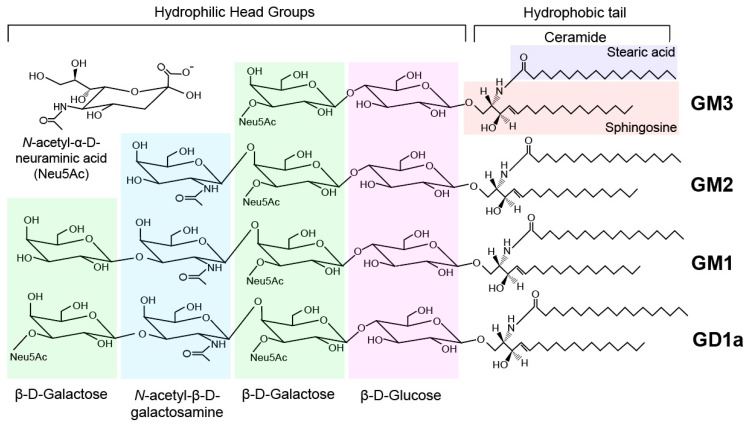
a-series ganglioside family structure similarity. All share the similarity of possessing a single sialic acid residue (shown as Neu5Ac) attached to the β-D-galactose (Gal) at position II. Note that ceramide ((2S,3R,4E)-2-(Acetylamino)-4-octadecene-1,3-diol) is composed of the sphingoid base, sphingosine (highlighted in orange), along with the saturated 18-carbon fatty acyl, stearic acid (highlighted in blue). Each ganglioside structure is divided based on its carbohydrate head group: β-D-glucose is the first sugar group, linked to the ceramide tail during the production of complex GSLs, shown highlighted in pink. β-D-glucose conjugated with ceramide alone would produce glucosylceramide (GlcCer); however, this is not depicted above. The addition of Gal and sialylation of the 3-OH of position of Gal (as highlighted in green) yields GM3 ganglioside. Note that although the green rectangle intends to highlight the Gal residues of gangliosides, Neu5Ac is conjugated to the second Gal of GM3, GM2, GM1, and GD1a ganglioside, as well as the terminal Gal residue of GD1a. The structure of the most common sialic acid in mammalian cells, *N*-acetyl-α-D-neuraminic acid (Neu5Ac), is depicted in the top left corner. The addition of *N*-acetyl-β-D-galactosamine (GalNAc) to the second Gal through a β-1,4 linkage produces GM2. The subsequent conjugation of a terminal Gal through a β-1,3 linkage to the GalNac moiety yields GM1, which can then be sialylated to form GD1a.

The defining structural characteristic of gangliosides are the sialic acid residues they may possess. Gangliosides are classified according to the number of sialic residues α2-3-linked to the galactose (Gal) structure at position II, where the 0-series gangliosides have zero sialic acid residues, the a-series possess one, the b-series have two, and the c-series have three sialic acid residues. The a-series gangliosides can be seen in Figure 1. Sialic acid may also be bound to N-acetylgalactosamine (GalNAc) in gangliosides via an α2-6 linkage, and the further addition of more sialic acid residues occurs through an α2-8 linkage. The type of sialic acid found in gangliosides can also differ between species, with over 50 organic members existing in this group of monosaccharides [33]. All sialic acids derive from the structure of 2-keto-3-deoxy-nononic acid (Kdn), a 5-amino derivative known as neuraminic acid, by means of acetylation or glycosylation at any of the non-glycosidic hydroxyl residues of Kdn. As seen in Figure 1, the major sialic acid of mammalian gangliosides is N-acetylneuraminic acid (Neu5Ac), a fairly strong acid (pKa of 2.6), which is a derivative of neuraminic acid that has been acetylated at position 5. It has been found that humans also possess, in very small quantities, the sialic acid derivative N-glycolylneuraminic acid (Neu5Gc) despite lacking the enzyme CMP-N-acetylneuraminic acid hydroxylase, which is needed to convert Neu5Ac to Neu5Gc [34]. Interestingly, it was discovered that almost all human ancestors express this enzyme [35]. The lack of enzyme expression in humans can be explained by the inactivation of the human CMP-N-acetylneuraminic acid hydroxylase gene (*CMAH*), causing the loss of the enzyme’s functionally critical N-terminus. It has been theorized that human brain expansion and evolution may have occurred following this inactivation [35,36]. Ganglioside structures differ in a stepwise fashion, such that each new ganglioside is the result of an additional step of glycosylation. For example, in the a-series gangliosides (Figure 1), LacCer is sialylated to form GM3. The addition of GalNAc to GM3 produces GM2 ganglioside, which can then form GM1 with the addition of Gal.

##### Glucuronoglycosphingolipids and Sulfatoglycosphingolipds

Glucuronoglycosphingolipids and sulfatoglycosphingolipids are negatively charged by means of a glucuronic acid or sulfate ester moiety, respectively. Glucuronic acid is a sugar acid derived from glucose with C6 oxidized to a carboxylic acid. To date, glucuronic acid-containing GSLs are not well studied as they are less frequently found in humans. In 1987, two acidic glycolipids were isolated from the peripheral nervous system of human patients. It was found that the isolated structures were sulfated glucuronic acid-containing paraglobosides that acted as receptors for IgM antibodies, causing peripheral neuropathy [37]. However, these structures are more prevalent in insect and plant species.

Sulfated GSLs are more commonly found in humans and are ubiquitously expressed in vertebrates, concentrated within the nervous system [38,39]. A common form of sulfatoglycosphingolipids is the structure sulfatide, which constitutes up to 4–6% of myelin lipids [40]. However, sulfatide has also been found in the kidney and small intestine [41,42]. Sulfatide is produced via esterification of a sulfate group, donated by 3′-phosphoadenosine-5′-phosphosulfate onto the 3-OH of Gal in GalCer. Sulfatides are readily deprotonated to form the HSO_4_^−^ ion. Similar to other GSLs, sulfatide structure can vary according to acyl chain length and differences in ceramide structure.

## 3. Sphingolipid Biosynthesis

### 3.1. Biosynthesis of Sphingoid Bases and Ceramide via the de novo Synthetic Pathway

The biosynthesis of ceramide, the precursor of complex sphingolipids, can be accomplished through either the *de novo* pathway, or the salvage pathway.

The *de novo* synthetic pathway begins in the endoplasmic reticulum (ER) with the decarboxylating condensation of the amino acid, L-serine, and an activated fatty acyl coenzyme-A (CoA). The most commonly used fatty-acyl CoA to form sphingolipids is palmitoyl-CoA (C16-CoA). However, the choice of acyl-CoA substrate is determined by the subunit composition of the SPT enzyme, which performs the condensation reaction [43,44]. SPT is a pyridoxal 5′ phosphate-dependent enzyme, which belongs to the A-oxoamine synthase family. This heterodimer is composed of two catalytic subunits (SPTLC1, SPTLC2), or alternatively, with a third regulatory subunit (SPTLC3) in place of SPTLC2 [43]. Additional protein families, small subunit SPTs (SPTssa and SPTssb), and orosomucoid-like proteins (ORMDLs) play important regulatory roles with regard to SPT complexes, leading to either increases (ssSPTs) or reductions (ORMDLs) in their activity [43,44]. The combination of the different subunits elicits a preference for specific acyl-CoAs, with palmitoyl-CoA (C16-CoA) being selected by the complex SPTLC1/SPTLC2/SPTssa. Both myristoyl-CoA (C14-CoA) and stearoyl-CoA (C18-CoA) are also used preferentially by SPTLC1/SPTLC3/SPTssa and SPTLC1/SPTLC2/SPTssb, respectively [10,45].

Mutations in the genes encoding the two primary subunits (SPTLC1, SPTLC2) result in substrate preference changes for the amino acids alanine and glycine in lieu of serine. The resultant non-canonical sphingolipid bases, 1-deoxysphinganine and 1-deoxymethylsphinganine, form ‘dead end’ ceramides, which due to the missing C1-OH moiety cannot be further modified [46]. The inability to progress in the sphingolipid metabolic pathway means that deoxysphingolipids cannot be degraded and accumulate to the point of inducing toxicity, as seen in hereditary sensory autonomic neuropathy-1 (HSAN1) [47,48,49,50,51,52,53].

**Figure 2:**
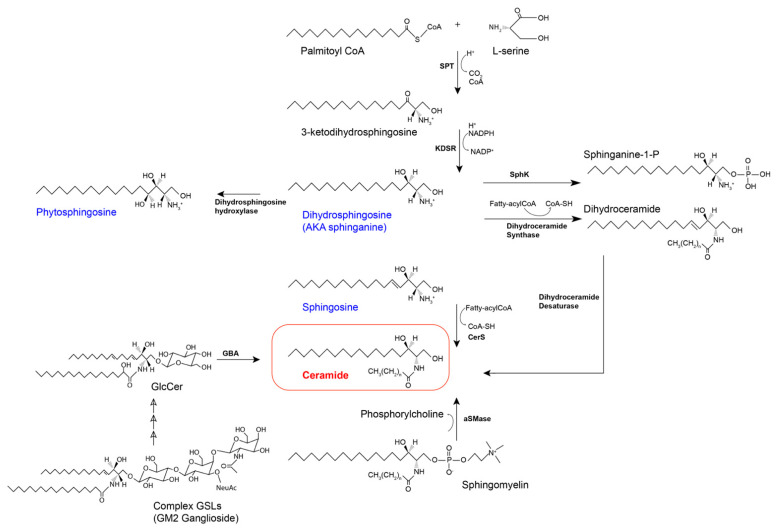
Summary of Ceramide Synthesis Pathways. A summarized schematic of the biosynthesis pathways which may produce ceramide, including the *de novo* pathway (right) and the salvage pathway (bottom). All sphingoid bases are depicted in blue. The *de novo* pathway begins with synthesis of the sphingoid base sphinganine and ends with ceramide desaturation. Sphinganine is also capable of producing phytosphingosine or sphinganine-1-phosphate. The salvage pathway consists of two streams: SM hydrolysis and GSL recycling. SM is converted to ceramide through the hydrolysis of the phosphocholine unit. The components of complex GSLs can also be recycled to reform ceramide through stepwise hydrolysis of carbohydrate units, eventually leaving a simple GSL such as GlcCer, depicted above. GlcCer is then converted to ceramide by glucocerebrosidase. GSL: glycosphingolipid; SM: sphingomyelin; aSMAase: acid sphingomyelinase; GlcCer: glucosylceramide; GBA: glucocerebrosidase; CoA: acyl coenzyme-A; CoA SH: coenzyme A; SphK; sphingosine kinase; CerS: ceramide synthase.

The product of the condensation of serine and palmitoyl-CoA is 3-ketodihydrosphingosine, which is then reduced by NADPH-dependent 3-ketodihydrosphingosine reductase (KDSR). The ketone located at C3 of 3-ketodihydrosphingosine is reduced into an alcohol, thus producing the amino alcohol sphinganine (dihydrosphingosine) [11,54]. Sphinganine can be converted into three different derivatives. The first, sphinganine-1-phosphate, is produced through ATP-dependent phosphorylation by sphingosine kinases [55]. Secondly, as seen in Figure 2, sphinganine can also be converted to phytosphingosine (4-hydroxysphinganine), with the addition of a hydroxyl group on C4.

The continuation of the *de novo* pathway creates a third biosynthetic avenue for sphinganine, which is the formation of dihydroceramide, as illustrated in Figure 2. Dihydroceramide synthase, more commonly referred to as ceramide synthase (CerS), catalyzes the attachment of an acyl group from a fatty acyl-CoA via amide linkage to the free amino group of sphinganine, producing dihydroceramide [10]. There are six enzymes in the CerS family which have been identified, each with a specific preference for the length of acyl-CoA chain used for N-acylation of the sphingoid LCB. CerS1, the first of the CerSs to be discovered due to its homology with Lag1 in yeast, preferentially uses C18-CoA. CerS2 prefers C22–C24-CoAs, CerS3 uses C26-CoA and higher, CerS4 uses C18-C22-CoAs, and CerS5 and CerS6 both primarily use C16-CoA [10,56,57,58]. The dihydroceramide produced by these sphingosine N-acyltransferases is then dehydrated by dihydroceramide desaturase, adding a 4, 5-*trans*-double bond, thus producing ceramide [59].

### 3.2. The Salvage Pathway

Complex sphingolipids, such as SM and GSLs, are partially degraded and their respective components are recycled to form ceramide in what is known as the salvage pathway, depicted in Figure 2. The hydrolysis of SM, producing ceramide and phosphocholine, by sphingomyelinases (SMases) is referred to as the sphingomyelinase pathway [30]. A more in-depth examination of SMases is provided in the catabolism section. GSLs are transported from the plasma membrane along endocytic routes to the lysosomes, where they are degraded by specific enzymes with the assistance of accessory proteins [60,61]. Following the removal of the glycan moieties, the remaining product is ceramide, which is then deacylated by ceramidases (CDases), producing sphingosine and a free fatty acid. Sphingosine is exclusively produced in the salvage pathway by the hydrolysis of complex sphingolipids and ceramide [62]. The phosphorylation of sphingosine by sphingosine kinases (SphK1 & SphK2) produces S1P. Alternatively, sphingosine can be transported to the ER, where it is re-used for the formation of ceramide, by CerS, and the subsequent production of complex sphingolipids [62].

### 3.3. Formation of Complex Sphingolipids

Complex sphingolipids are formed by attaching a hydrophilic head group to the hydroxyl group located at C1 of a hydrophobic ceramide. As previously mentioned, they can be divided into two categories: PSLs and GSLs. Ceramide, produced by either the *de novo* pathway or the salvage pathway, is the foundational base for all complex sphingolipids.

#### 3.3.1. Phosphosphingolipids

PSLs are formed through the attachment of a phosphate-containing polar head group to the ceramide parent compound, which in the case of SM is phosphocholine [63]. Following the completion of ceramide synthesis in the ER, it is then transported to the inner leaflet of the Golgi apparatus by the ceramide transport protein (CERT) [60,64,65]. A phosphocholine head group is enzymatically transferred from a phosphatidylcholine to the ceramide by a sphingomyelin synthase (SMS), producing diacylglycerol (DAG) and SM (ceramide phosphocholine) [30]. There are three sphingomyelin synthases (SMS1, SMS2, and SMSr) encoded by the genes *SGMS1*, *SGMS2*, and *SAMD8,* respectively. SMS1 and SMS2 each have six transmembrane domains and perform the same catalytic function, but are located in different sites; SMS1 is found in the trans-Golgi apparatus, whereas SMS2 is located in the plasma membrane [30,66].

As an alternative to the choline used to form SM, ethanolamine can be used as the phospho-alcohol portion, provided by a phosphatidylethanolamine to produce ceramide phosphoethanolamine (CPE). The less active homolog to SMS, known as sphingomyelin synthase-related protein (SMSr), preferentially uses phosphatidylethanolamine as a donor, thus producing CPE [67]. Like its SMS homologs, SMSr is a six-transmembrane protein, though is located in the ER lumen [68].

Sphingosine, which is generated by the salvage pathway, is phosphorylated at the C1 hydroxyl group by a sphingosine kinase to form S1P. This pathway can occur in the plasma membrane, mitochondria, nucleus, and in lysosomes. There are two forms of sphingosine kinase, SphK1 and SphK2. SphK1 is primarily located in the cytosol and phosphorylates sphingosine that has been exocytosed from lysosomes to form S1P [69]. However, SphK1 can be phosphorylated by extracellular signal-regulated kinase 1/2 (ERK1/2), at which point it moves to the plasma membrane, where it likewise forms S1P with the sphingosine in the membrane [69]. Meanwhile, SphK2 is localized primarily to the nucleus and mitochondria [64,70,71].

C1P is produced by the direct phosphorylation of ceramide by a ceramide kinase (CERK), typically in the *trans*-Golgi networks, though it is also found in the nucleus and plasma membrane. CERK contains an N-terminal myristoylation site and pleckstrin homology domain, used for cell membrane association [64,72], and is a member of the DAG kinase family [30]. CERK preferentially targets sphingosine-containing ceramides, with further specificity towards those with acyl chain lengths longer than 12 carbons [30]. Following its production, C1P is transported to the plasma membrane by the ceramide phosphate transfer protein (CPTP) [64].

#### 3.3.2. Glycosphingolipids

GSLs are formed through the conjugation of a hydrophobic ceramide base and a hydrophilic carbohydrate head group, which can be broadly classified based on their carbohydrate composition into neutral and acidic GSLs. Neutral GSLs are also commonly referred to as cerebrosides.

**Figure 3:**
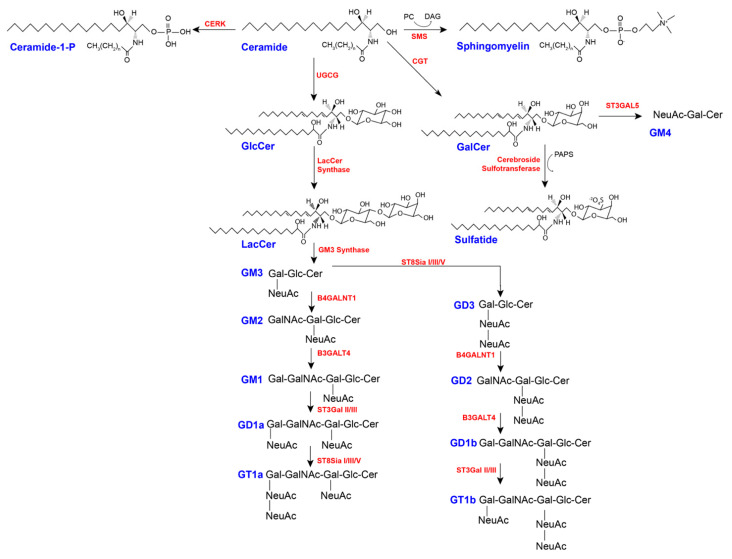
Summary of Complex GSL Biosynthesis. Ceramide is a major branching point in the biosynthetic pathway for several structures, including SM, C1P, and the simple GSLs GlcCer and GalCer. Ceramide glucosyltransferase (UGCG) is responsible for the addition of a glucose molecule to ceramide, whereas ceramide galactosyltransferase (CGT) adds a galactosyl molecule to ceramide. GalCer can be sialylated by the sialyltransferase ST3Gal V to produce GM4. In addition, GalCer may be sulfated by cerebroside sulfotransferase to form sulfatide. GlcCer is converted to LacCer by the addition of Gal onto the Glc head group. LacCer is another major branching point for complex GSL biosynthesis; however, only two synthetic pathways are illustrated above, which are the a- and b-series gangliosides (left and right, respectively).

As seen in Figure 3, once formed in the ER, ceramide can be galactosylated by the enzyme ceramide galactosyltransferase (CGT), a type I transmembrane protein, using uridine diphosphate galactose (UDP-Gal), forming GalCer on the luminal face of the ER [60,73]. GalCer can then be transported to the Golgi for further modification, such as the addition of sulfate to the C3 hydroxyl group, turning it into sulfatide, or it can be sialylated by the sialyltransferase ST3GalV to form Neu5Acα2-3GalβCer (GM4) [30]. Alternatively, ceramide can follow one of two trafficking pathways to the Golgi complex: (1) transportation via CERT, which delivers ceramide to the *trans*-Golgi network for development of SM; or (2) vesicular transport to the *cis*-Golgi network, for the production of GlcCer through glycosylation [60,65]. The formation of GlcCer is mediated by the enzyme UDP-glucose ceramide glucosyltransferase (UGCG), which transfers one glucose taken from activated UDP-glucose to the hydroxyl group at C1 of the ceramide in β-linkage (*O*-linked glycosylation) [74,75].

GlcCer is transported through the Golgi and can be galactosylated by β4-galactosyltransferases V and VI, forming LacCer, which becomes a branching-off point for either the addition of more monosaccharides, forming globosides (neutral GSLs), or the addition of one or more acids and the subsequent development of acidic GSLs. These different groups formed from the LacCer substrate are considered the GSL series and are clustered based on a shared neutral tetrasaccharide core. There are seven GSL series, the most important for vertebrates being the ganglio-, globo-, and neolacto-series. The formation of GSL series from the same LacCer substrate is dependent on the availability and intracellular distribution of different Golgi glycosyltransferases [75]. Glycosyltransferases interact with each other, sometimes resulting in their differentiation to different Golgi subcompartments. Such is the case with LacCer synthase, which physically interacts with GM3 synthase and globotriaosylceramide (Gb3) synthase, resulting in its relocation to a different subregion. Therefore, the metabolic outcome of GlcCer is dependent on where in the Golgi it is delivered [76]. The glycosyltransferases which produce the precursors for the ganglio-, globo-, and neolacto-series, respectively, are as follows: GM3 synthase (lactosylceramide α-2,3-sialyltransferase), encoded by the gene *ST3GAL5*, produces GM3 (NeuAcα2-3Galβ1-4Glcβ1Cer); Gb3 synthase (lactosylceramide α1-4-galactosyltransferase), encoded by the gene *A4GALT*, produces Gb3 (Galα1-4Galβ1-4Glcβ1-Cer); and Lc3 synthase (lactosylceramide β -1,3-*N*-acetylglucosaminyltransferase), encoded by the gene *B3GNT5*, produces Lc3 (GlcNAcβ1-3Galβ1-4Glcβ1Cer) [60].

## 4. Sphingolipid Catabolism

### 4.1. Degradation of Complex Sphingolipids

Similarly to the stepwise production of sphingolipids during biosynthesis, their catabolism follows the reverse path, with products now acting as the substrates. Improper hydrolysis of sphingolipids results in a number of diseases. The exploration of these diseases lead to the discovery of the majority of the responsible lysosomal hydrolyses and accessory proteins, as well as knowledge regarding their genetic origins.

Complex sphingolipids are removed from the plasma membrane and taken to the lysosomes for degradation via the endolysosomal pathway. [61,77,78,79]. Specific features of the membranes of late endolysosomal compartments allow for the protection of the organelle during the degradation of membrane lipids. A glycocalyx forms a protective layer on the luminal side of the endolysosomal perimeter membrane, which protects it from deterioration as the interior pH value decreases in preparation for lipid degradation [61]. Formed in late endosomes, just prior to fusion with lysosomes, bis(monoacylglycerol)phosphate (BMP), an anionic lipid, is a main activator for enzymatic sphingolipid degradation [80]. BMP is located only on the luminal side of late endosome and intralysosomal membranes due to its unique sn1:sn1′ stereoconfiguration. In combination with dolichol phosphate and phosphatidylinositol, BMP creates a negative charge in the intralysosomal membranes. This charge attracts activator proteins and hydrolytic enzymes, which have a positive charge at an acidic pH, leading to their adherence to the membrane, where they can then degrade the sphingolipids located there [61].

#### 4.1.1. Sphingomyelin Breakdown

As the most abundant of the sphingolipids, SM catabolism is particularly important for the cell. The catabolism of SM is mediated by a family of three SMases, which are grouped based on their optimal pH: acidic, neutral, and alkaline. These enzymes hydrolyze the phosphocholine head group from SM, producing ceramide and free phosphocholine. Acid sphingomyelinase (aSMase), from the gene *SMPD1*, is a precursor protein which is then modified through the addition of mannose oligosaccharide residues. After modification, aSMase is trafficked into either a lysosomal or secretory pathway, becoming either lysosomal aSMase (L-aSMase) or secretory aSMase (S-aSMase). L-aSMase acquires multiple mannose-phosphate residues through the actions of *N*-acetylglucosamine-1-phosphotransferase and *N*-acetylglucosamine phosphodiesterase on the mannose residues of the aSMase precursor. L-aSMase is shuttled to endolysosomes via vesicles containing mannose-phosphate receptors, where it then catabolizes SM located on the endosomal membranes [30,81]. aSMase that is not mannose-6-phosphorylated is trafficked through the Golgi secretory pathway to the extracellular space. S-aSMase is zinc-dependent, an aspect thought to be lost in L-aSMase due to exposure of cellular zinc [30,81]. The structure of aSMase includes a catalytic domain and a saposin-like domain [61]. A paralogue of aSMase, sphingomyelin phosphodiesterase acid-like 3b (SMPDL3b), encoded by the gene *SMPDL3B*, has been shown to dephosphorylate another of the PSLs, C1P. Binding specifically to C16-C1P, SMPDL3b reduces C1P to ceramide [82,83].

Four neutral sphingomyelinases (nSMase) have been identified in mammals, nSMase1, nSMase2, nSMase3, and MA-nSMase (mitochondrial-associated nSMase), encoded by the genes *SMPD2*, *SMPD3*, *SMPD4*, and *SMPD5*, respectively. These nSMases are magnesium-dependent and share a common catalytic core similar to DNase I-type, with the exception of nSMase3. The most common is nSMase2, which is localized to the Golgi apparatus and plasma membrane [84]. nSMase2 translocates from the Golgi to the plasma membrane following stimulation from various factors, including TNF-α, H_2_O_2_, and PMA [84,85,86]. Alkaline sphingomyelinases (alk-SMases), encoded by the gene *ENPP7*, were discovered to catalyze the hydrolysis of SM into ceramide at an optimal alkaline pH. However, these enzymes were later discovered to have no structural similarities to acid or neutral SMases. Instead, alk-SMases share almost 30% similarity to the amino acid sequences of the enzyme family ecto-nucleotide pyrophosphatase/phosphodiesterase (NPP), thus gaining the synonymous name NPP7 [87]. Alk-SMases are only found in the liver and intestines, where they play an important role in the breakdown of dietary SM [30].

#### 4.1.2. Glycosphingolipid Breakdown

GSLs in the intralysosomal membrane with short carbohydrate chains (four or fewer sugars) are not easily accessible to their hydrolyzing enzymes and require the additional assistance of lysosomal lipid binding proteins (LLBP). These auxiliary proteins include the GM2 activator protein (GM2AP), encoded by the *GM2A* gene, and the four saposins (A–D), which are derived from a common precursor protein, prosaposin (*PSAP*), via proteolysis [61,88]. The role of GM2AP and saposins is to bind, solubilize, and present GSLs for degradation by their respective hydrolases [61]. The structures of the four saposins are very similar but they differ in specificity and in how they activate lysosomal hydrolyses [88].

The majority of glycosyl hydrolases (GHs) were identified while exploring the causes of various LSDs. As with their biosynthesis, the lysosomal catabolism of GSLs follows a strict sequential pathway. In the ganglio-series (a), GM1-β-galactosidase, encoded by the *GLB1* gene, catabolizes GM1 by cleaving the terminal β-D-galactose from GM1 with the assistance of either GM2AP or saposin B, producing GM2 [61,89,90]. The heterodimeric enzyme β-Hexosaminidase A (HexA), encoded by genes *HEXA* and *HEXB*, works in conjunction with GM2AP to remove the terminal β-glycosidically linked N-acetylgalactosamine (GalNAc) from GM2, further reducing them to GM3 [91]. HexA and its two isoforms (B and S) also degrade other glycolipids, such as GA2 from the asialo-series and globosides with N-acetylhexosamine residues [61,92]. GM3 hydrolysis is catalyzed by sialidase (α-N-acetyl neuraminidase), which is encoded by the gene *Neu1*. Saposin B works with the sialidase to remove the terminal sialic acid residues from GM3, producing LacCer [93]. The hydrolysis of LacCer can be catalyzed by either GM1-β-galactosidase with saposin B or by galactosylceramide-β-galactosidase, encoded by the gene *GALC,* with saposin C [94,95]. The GlcCer produced from LacCer hydrolysis is hydrolyzed by glucosylceramide-β-glucosidase, encoded by the gene *GBA*, assisted by saposin C, producing ceramide. Sulfatides are catabolized by arylsulfatase A (*ARSA* gene) with saposin B, generating GalCer [95]. Galactosylceramide-β-galactosidase is also responsible for the degradation of GalCer to ceramide through the removal of its Gal, with saposin A as the accessory protein, creating ceramide.

### 4.2. Ceramide Catabolism

All complex sphingolipids are broken down to their parent compound, ceramide, which is then in turn catabolized into sphingosine, which can be phosphorylated to form S1P or recycled in the salvage pathway to produce new ceramide.

The deacylation of ceramide to produce sphingosine and free fatty acyl-CoAs is performed by a family of hydrolyses known as ceramidases (CDases). Five CDases have been characterized, and like the SMases, they are grouped by the optimal pH for their activity. Acid ceramidase (aCDase), encoded by the gene N-Acylsphingosine Amidohydrolase 1 (*ASAH1*), begins as a polypeptide which then self-cleaves into two subunits, forming a mature heterodimeric enzyme [96]. This lysosomal hydrolase preferentially catalyzes the hydrolysis of small-to-medium-chain ceramides (C6–C18). The neutral ceramidase (nCDase), encoded by the gene *ASAH2,* has two human isoforms. nCDase uses both ceramide and dihydroceramide as a substrate, and as a transmembrane glycoprotein it is found in various cellular compartments, including the plasma membrane. This enzyme is highly expressed in intestines, where it plays an important role in the breakdown of dietary ceramides. It also has a preference for C16 and C18 ceramides. The remaining three CDases are all alkaline ceramidases (alkCDases 1–3) and are encoded by the genes *ACER1*, *ACER2*, and *ACER3*, respectively. The smallest of the CDases, alkCDases, are found primarily in the Golgi apparatus and ER and preferentially degrade long-chain ceramides (C20–C24) [97]. Intriguingly, other transmembrane enzymes have been implicated in the catabolism of ceramides. A study by Vasiliaukaité-Brooks et al. (2017) showed that ADIPOR2, a membrane protein which mediates adiponectin, intrinsically binds with C18 ceramide and converts it to sphingosine and free fatty acyl. To a lesser extent, short (C6)- and long (C24)-chain ceramides are also broken down by ADIPOR2, but C18 appears to be the preferred ceramide substrate [98].

### 4.3. Sphingosine-1-Phosphate—The Final Breakdown

Sphingosine, produced by the hydrolysis of ceramide, can either be recycled through the salvage pathway or phosphorylated by SphK 1 and 2, with the assistance of ATP, to form S1P. S1P can be dephosphorylated in the plasma membrane by lipid phosphate phosphatases (LPP1-3), or cytosolic S1P can be dephosphorylated at the ER courtesy of S1P-specific phosphatases (SPP1 and SPP2). Alternatively, S1P can be further (and irreversibly) degraded by sphingosine-1-phosphate lyase (SPL) into hexadecenal and phosphoethanolamine [30]. SPL is a transmembrane protein localized exclusively to the ER, where it only has access to cytosolically produced S1P, as its catalytic site faces the cytosolic face of the ER. SPL is able to catabolize all mammalian sphingoid bases and is dependent on a cofactor, pyridoxal 5′-phosphate (PLP), for enzymatic activity [99].

## 5. Sphingolipid Functions

### 5.1. Membrane Domains and Signalling

The existence of microdomains, or membrane rafts, has been thoroughly debated over the years, likely due to the heterogeneity of their composition. Located in the outer leaflet of the plasma membrane and some intracellular membranes (ER and mitochondria), rafts are described as dynamic; small (10–200 nm); cholesterol-, sphingolipid-, and protein-rich domains that compartmentalize cellular processes [100,101]. The proteins associated with lipid rafts belong to various distinct classes, including signalling proteins (Src-family kinases), G-protein-coupled receptor proteins (GPCR), true resident proteins (caveolin, glycosylphosphatidylinositol-linked proteins), and palmitoylated (hedgehog) and myristoylated proteins [101,102]. Two types of rafts have been defined, caveolae and planar lipid rafts (aka non-caveolar). The caveolae domains have been morphologically defined as small invaginations (50–100 nm) with a characteristic flask-shape in the membrane, produced by polymerization of the transmembrane, where palmitoylated caveolin proteins bind tightly with cholesterol [103,104]. Planar lipid rafts are not as morphologically distinguishable, however, and are described as non-invaginated, continuous membrane domains [101].

The characteristic liquid-ordered phases of lipid rafts are a result of the saturated acyl chains of the sphingolipids, which readily fit closely together with cholesterol, creating a more viscous domain than the less-dense (liquid-disordered) phase seen in the majority of the plasma membrane [105,106,107]. The structure of individual sphingolipid species affects the fluidity, structure, and permeability of the membranes in which they reside [108,109]. Segregation of certain groups of sphingolipids has been attributed to an inter- and intramolecular hydrogen-bond network established by the hydroxyl and amine groups functioning as hydrogen-bond donors and acceptors [110,111,112]. A study by Santos et al. (2020) compared canonical sphingolipids to their derivatives, deoxysphingolipids, and found that the fluidity of the membrane is affected by the unsaturation of the LCB and the position structure of C1 (the presence or absence of methyl and hydroxyl groups). However, more significantly, the location of the double bond—4,5-*trans* in canonical sphingolipids; 14,15-*cis* in deoxysphingolipids—had a greater impact on membrane properties [19]. The increased distance from the polar head group of the 14,15-*cis*-double bond in deoxysphingolipids impairs the formation of ordered domains by increasing the area of the molecule and therefore its required space. The result of this is weakened interactions and hydrogen bonds between molecules due to the increased distance between them [111].

Both cholesterol and sphingolipids, particularly SM and GSLs, are found in high quantities in membrane rafts of the cells in the nervous system [113]. In fact, it has been demonstrated that increased concentration of lipid rafts in the membrane leads to more acyl chains and decreased motion, but increased lateral mobility; however, the latter point is still debated [114,115].

The functions of receptors and ion channels in microdomains are regulated by the composition of the microdomain, with both direct (lipid–protein interactions) and indirect factors (changes to the physical membrane) affecting them. Some of the changes to the domains are related to trafficking, membrane localization, and alterations in kinetics; however, different receptors and ion channels respond to microdomain regulation in different manners. For example, it was found that in oocytes, SM regulates the activity of the Kx2.1 channel by interacting with the helix-turn-helix motif found in the S3b and S4 voltage-sensing domains of the channel [116,117]. Proteins found in microdomains tend to be acylated, palmitoylated, or myristoylated, as a result of the preference for molecules with unbranched and saturated side chains within the tightly packed domain [118,119]. Acylated kinases, such as the Src-family of tyrosine kinases, in the microdomains are able to modulate those ion channels which are regulated by phosphorylation [78,120].

Ceramides are an important component for the structural stability of cell membranes as they are found in a high volume in the caveolae, where they contribute to cell signalling. Ceramides are known to be involved in cell differentiation, the promotion of inflammatory responses, and the induction of cell cycle arrest and apoptosis [121]. Ceramides can activate serine/threonine protein phosphatases [122,123], inhibit phospholipase D [124], and stimulate certain serine/threonine kinases [125], all of which modulates the signal transduction processes within microdomains [121].

GSLs are highly expressed in membrane domains and facilitate a variety of functions. LacCer constitutes more than 70% of the GSLs found in human neutrophils and clusters to form microdomains with signal transducer molecules in the plasma membrane. These LacCer-membrane rafts can bind with different species of pathogenic yeast and bacteria, which induces chemotaxis and phagocytosis in neutrophils, indicating an important role for LacCer in bactericidal functions [126]. A study by Iwabuchi et al. (2015) found similar LacCer-mediated phagocytosis and migration in mouse neutrophils.

Sphingolipids are known to be involved in cell–cell signalling; one example of this comes from a study which found that ceramide and nSMase are involved in the formation and secretion of exosomes which are implicated in cell–cell communication [127]. Additionally, inhibition of nSMase2 has been found to interfere with the cargo composition of exosomes (miRNA, prions, etc.) [128,129,130]. Meanwhile, the inhibition of SMS2 in neurons increases exosome secretion [130]. In vesicular trafficking, a specific species of SM (C18 SM) has been implicated due to its recognition by a component of COPI, a vesicular coat protein (coatomer) [46,131]. GM1 situated in membrane rafts can function as a receptor for multiple molecules, including prions and the cholera toxin (CT) [132,133]. CT binds and forms a receptor–toxin complex with GM1 in lipid rafts, which is then endocytosed and transported to the ER. In a study by Jobling et al. (2012), it was determined that modified holotoxins with only one or two GM1 binding sites were able to efficiently intoxicate host cells. This was contrary to the previous notion that pentameric binding would be required, as had been demonstrated by Ewers et al. (2010) with simian virus 40 (SV40) [134]. The association of SV40 with GM1 created invaginations in the PM, followed by endocytic transport to the nucleus of the host cell. Jobling’s study with CT also brought to light a subset of GM1 species with unsaturated ceramide domains which efficiently translocate from the PM to the ER, with the assistance of actin and flotillin, which are lipid-raft-associated proteins. The results of this study suggests that ceramide structure is an important component of protein-dependent mechanisms in lipid sorting [133].

### 5.2. Cell Death and Proliferation

Ceramide has been well established as a mediator of cell death, which was first identified as having a regulatory role in apoptosis and cellular senescence [3]. The vast number of ceramide species has made determining the exact mechanism involved in ceramide-mediated apoptosis more challenging [25]. Interestingly, certain species, such as CerS6-generated C16 ceramide, have been found to have a pro-survival function, and in one study, very-long-chain ceramides (C24, C24:1) were found to promote cell proliferation [3]. Meanwhile, species like CerS1-generated C18 ceramide have a pro-apoptotic role, and long-chain ceramides (C16, C18, C20) have been found to be antiproliferative [3,135].

Ceramide and S1P, the ‘sphingolipid rheostat’, work in opposition to each other as determinants of cell fate. S1P has been implicated in many functional roles, including cell proliferation and mitogenesis, and has demonstrated anti-apoptotic properties. S1P works as an antagonist to ceramide-mediated apoptosis by activating ERK and suppressing c-Jun N-terminal kinases (JNK) activation induced by ceramide [136,137]. Further exploration into cell fate effects associated with S1P have shown that SphK1 and SphK2 have opposing roles in the regulation of cell fate, with SphK1 being associated with antiapoptotic and mitogenic effects, whereas SphK2 has a pro-apoptotic effect [138,139]. In recent years, S1P has gained more attention from scientists as an important physiological mediator of homeostasis, particularly in the nervous, immune, and vascular systems [140]. Recently, S1P has been exploited as a drug target for several neurological disorders due to its cell survival properties [141]. S1P exerts its functions via two methods: (1) it is exported out of the cell for paracrine interaction, typically with the S1P receptor, and (2) it interacts with targets within the cell (reviewed in [142]). As S1P is a charged sphingolipid, it cannot readily cross the cell membrane and requires the aid of membrane transporters [142].

C1P was first proven to stimulate DNA synthesis and proliferation in fibroblasts, in addition to regulating cell growth in primary photoreceptor progenitors, primary bone marrow-derived macrophages (BMDM), C2C12 macrophages, and various cancer cell types [121]. The stimulation of cell proliferation by C1P involves multiple signalling pathways. For example, in BMDM, C1P activates the ERK, JNK, and PI3-K/PKB pathways and phosphorylates NF-κB and GSK-3β, which then targets cyclin D1 and c-Myc, both of which are upregulated by C1P. The result of this process stimulates the proliferation of macrophages [121,143]. Additionally, the phosphorylation of the mammalian target of rapamycin (mTOR), specifically part of the kinase complex (mTORC1), has been found to be a crucial step in the mechanism by which C1P stimulates the proliferation of macrophages [144].

Gangliosides are omnipresent in mammalian cell membranes, and their role in cell differentiation and proliferation has been well examined. Gangliosides modulate and regulate various growth factor receptors (GFR), including tyrosine kinase GFRs, platelet-derived GFRs, fibroblast GFRs, and epidermal GFRs [145]. Studies have shown that GM3 works as a physical modulator of epidermal GRF (EGFR), by acting as a physiological competitor for dimerization, GM3 inhibits EGFR autophosphorylation, resulting in the inhibition of cell proliferation and growth [145]. GM3 has also been implicated in the development of insulin/IGF-1 resistance in keratinocytes, and Dam et al. (2017) found that GM3 depletion activates the IGF-1R/Rac1 pathway, which promotes keratinocyte migration [146].

### 5.3. Cell Migration and Invasiveness

The role of sphingolipids in cell migration and invasiveness is complex and has important implications in pathophysiology, as discussed later in this paper.

A role for S1P as an activator for the ezrin, radixin, and moesin (ERM) family of proteins was elucidated by Adada et al. (2015) [147]. ERM proteins have previously been demonstrated to regulate cell mobility and have an influence on cancer invasion. In a study by Adada et al. (2015), the authors found that SphK2 plays a critical role in EGF-mediated ERM phosphorylation, resulting in increases in ERM activity, in HeLa cells [147]. Knocking down SphK2 produced a decrease in ERM activity, whereas its overexpression resulted in increases in EGF-mediated invasion and adhesion due to increased activity in ERM proteins [147,148]. Complementing this finding, ceramide plays a role in the activation of the protein phosphatase PP1alpha, which dephosphorylates ERMs, thereby reducing their activity [46,122].

C1P also has been found to promote cell migration through the activation of the transcription factor NF-κB and the PI3K/Akt and MEK/ERK1-2 pathways by interacting with a receptor coupled to G proteins in the plasma membrane [121]. This activation was found to be crucial for the migration of macrophages in a study using pertussis toxin, which blocked G proteins [121,149].

### 5.4. Inflammation

The accumulation of ceramide has been shown to induce inflammatory oxidative stress in cystic fibrosis (CF) and emphysema [150]. Ceramide was thought to be implicated in neuroinflammation due to its association with apoptosis. This was confirmed in a study by De Wit et al. (2019), who found increased levels of ceramide in reactive astrocytes, and consequently an elevation in neuroinflammation [151].

S1P is commonly implicated in inflammatory roles; one such example is from a study by Yogi et al. (2011), which showed that S1P promotes activation of p38MAPK and JNK/SAPK, and induces inflammatory mediators, in addition to stimulating inflammatory pathways through receptor tyrosine kinase phosphorylation, mediated by S1P1 receptors [152]. This response was amplified in spontaneously hypertensive stroke-prone (SHRSP) rats, likely due to the increased phosphorylation of platelet-derived growth factor and epidermal growth factor receptor. The results of that study indicate that S1P induces proinflammatory signalling pathways that may affect vascular inflammation in hypertension [152]. Additionally, elevated proinflammatory responses, including elevated proinflammatory cytokines (IL-23/IL-17/G-CSF cytokine axis), increased gene expression for inflammatory products, and elevated blood neutrophils and monocytes, were found in an S1P lyase knockout mouse model as a result of S1P accumulation [99]. Conversely, evidence for S1P in an anti-inflammatory role has been demonstrated in a study by Fettel et al. (2019), which showed that S1P suppressed leukotriene (LT) biosynthesis in human neutrophils [153]. LTs are inflammatory mediators. S1P interrupted the biosynthetic pathway by inducing S1P4-mediated Ca^2+^ mobilization, followed by 5-lipoxygenase (5-LO) translocation, and finishing with the irreversible inactivation of 5-LO, which normally catalyzes the synthesis of LTs [153].

Both S1P and C1P have been implicated in intestinal inflammation following abdominal surgery. Rat intestinal smooth muscle cells that were incubated with both sphingolipids exhibited elevated production of PGE_2_. S1P on its own enhanced cyclooxygenase 2 expression, whereas C1P caused an increase in arachidonic acid (AA), which is freed from membrane phospholipids by phospholipase A_2_ activity (PLA2); therefore, increases in AA indicate enhanced PLA2 activity [154]. A study by Pettuss et al. (2003) also found evidence for C1P as a major regulator of the inflammatory response via activation of AA and prostaglandin synthesis and release [155]. They also identified CERK, which produces C1P, as an upstream regulator of PLA2 activation [155].

### 5.5. CNS Development

The lipid-rich environment of the nervous system is home to an abundance of various sphingolipids, which play critical roles in the development and maintenance of the nervous system. The type and distribution of sphingolipids in the CNS is specific not only to distinct cell types, but to regions of the CNS as well. Myelin and oligodendrocytes are highly enriched by SM and GSLs, such as GalCer and sulfatide, whereas high quantities of gangliosides are found in neurons and grey matter [78,156,157]. Throughout human CNS development the sphingolipid profile dramatically changes, indicating their role in differentiation [78].

During the first two years of postnatal development there is a marked change in the type of SM found in myelin, with a notable increase in the amount of long-chain fatty acyl (C24:0 and C24:1) SMs, balanced by a decrease in medium-chain SMs (C18). The exception to this is the cerebral cortex, where the ratio of SM stays relatively stable in the first two years of life [78,158]. Likewise, there is a remarkable change in the gangliosides, which comprise 10–12% of the membrane lipid content in grey matter during development and into adulthood [159]. The strictly regulated and region-specific changes to ganglioside expression are suspected to direct brain maturation. These changes in expression typically occur in tandem with certain neurodevelopmental milestones, such as neuronal differentiation, axonogenesis, dendritic outgrowth, and synaptogenesis, all of which are correlated with an increase in GD1a and GM1 [78]. A study by Dasgupta and Ray (2017) identified the peak moments of participation of several sphingolipids in myelination during CNS development in rats [160]. Ceramide and dihydroceramide levels were highest in the embryonic stage and GlcCer was highest in the early postnatal stage, with GalCer not appearing until postnatal day 10. A similar trend was seen with sphingosine and sphinganine, with an initial peak at day 10, followed by lower levels by day 21. These results indicate the importance of various sphingolipids at specific stages of rat brain development [160].

## 6. Sphingolipids’ Role in Pathophysiology

Sphingolipids have proven to be crucial signalling molecules in many pathophysiological processes involved in inflammation, cancer, metabolic diseases, neurodegenerative disorders, LSDs, and others. Ceramide, C1P, S1P, and other sphingolipids are widely studied and have unique but integral roles in many of the previously stated pathologies. Here, we will discuss some of these processes.

### 6.1. Inflammatory Diseases

Sphingolipids have an essential role in the regulation of the inflammatory response, which primarily contributes to conditions like CF, asthma, and inflammatory bowel disease (IBD) (summarized in Table 2). Teichgräber et al. (2008) identified ceramide as a key regulator of inflammation and infection in CF mice [161]. Deficiency of *Cftr*, the gene encoding the cystic fibrosis transmembrane conductance regulator, which is linked to CF, indirectly results in an accumulation of ceramide in intracellular vesicles, caused by the alkalinization of vesicles in respiratory cells leading to an imbalance of aSMase and aCDase [161]. ASMase is now being used as a target for potential therapeutics of CF [161,162]. Additionally, a study by Bodas et al. (2015) found that LacCer, which accumulates as a result of exposure to cigarette smoke, leads to aberrant autophagy and apoptotic-inflammatory response, contributing to severe emphysema in patients with chronic obstructive pulmonary disease (COPD) [163].

Furthermore, sphingolipids have also been linked to inflammation in asthma (deficiency in the *ORMDL3* gene) [164,165], a condition characterized by bronchial hyperresponsiveness and proliferation of airway smooth muscle cells [166]. S1P acts as a positive regulator for the mast cell response through calcium mobilization [167], both intra- and extracellularly [168], through “inside-out” signalling [169]. S1P binds GPCR on the surface of surrounding cells [170] to transactivate S1P receptors 1 and 2, which are involved in mast cell migration and degranulation, respectively [171]. It was previously thought that S1P also played a role in acute lung injury; however, these data were unable to be replicated and thus more research is required on this topic.

Moreover, many sphingolipids play a crucial role in the proinflammatory response observed in IBD, likely triggered by mutations in *IL6* (encoding interleukin6) [172]. SM and ceramide levels were found to be significantly increased in experimental models of Crohn’s disease and chronic colitis. SM agglomeration can be explained by a decrease in the neutral and alkaline isoforms of SMase, as well as an increase in SMS2 [173,174,175]. Moreover, the accumulation of ceramide is elucidated by an increase in GlcCer and ceramide synthase, which is commonly found in IBD patients [176]. Lastly, sphingosine, S1P, and C1P are also elevated in IBD and can be attributed to the increased activity of nCDase and the enhanced expression of SphK1, respectively [177,178]. Each of these enzymatic alterations function to increase the levels of sphingolipids in various parts of the gastrointestinal tract, consequently playing a role in the associated inflammation seen in various forms of IBD.

### 6.2. Cancer

Similar to other pathologies discussed in this section, alteration in sphingolipid metabolism is also associated with various cancers (summarized in Table 3). Sphingolipids act as secondary messengers to cancer cell signalling pathways that regulate growth, proliferation, migration and/or metastasis. Many studies suggest that disruption of the innate S1P:ceramide ratio plays a pivotal role in regulating cancer cell death and survival. A previous review highlighted the role of sphingolipid metabolism in cancer [179]; here, we will summarize those findings.

#### 6.2.1. Ceramide Synthesis

Ceramide species are differentially regulated by CerS depending on tissue and cell type, suggesting that the role of ceramide varies between cancer types. CerS1, which generates C18 ceramide, was found to be inhibited in head and neck cancer cells through histone deacetylase 1 (HDAC1) and mi-R-574-5p [180]. More specifically, HDAC1 and mi-R-574-5p regulate CerS1 mRNA by targeting the 3′UTR of mRNA for rapid degradation, thus disrupting expression [181]. Upon returning C18 ceramide levels to concentrations similar to that in normal head and neck tissues, cell growth was inhibited by approximately 70–80% due to the modulation of telomerase activity and the induction of apoptotic cell death by mitochondrial dysfunction [181]. Interestingly, CerS6 has the opposite effect in squamous cancer cells by protecting the integrity of the ER and Golgi membranes and preventing downstream activation of activating transcription factor 6, which induces stress-mediated apoptosis [135,182]. Knockdown of CerS6, through siRNA, induced activating transcription factor 6 and apoptosis in multiple human cancer cells via the perturbation of calcium levels [135,182]. However, a recent study demonstrated that transcriptional activation of CerS6 through p53 activates ceramide biosynthesis, which contributes to a pro-apoptotic cellular response in adenocarcinoma cells [183]. Furthermore, 50% of mice with the loss of CerS2 developed a pheochromocytoma, a benign tumor of the adrenal gland, likely explained by the inability to synthesize ceramides with very-long (C22–C24) acyl chains [184].

The hydrolysis of SM to ceramide plays a role in cancer development through the altered expression of SMases. In human lymphoblasts, induction of aSMase expression resulted in apoptosis due to increased ceramide generation [185,186,187]. However, another study found that when *Smpd1* (encoding aSMase) was knocked out, mice were protected from lung and spleen metastasis by melanoma cells and when wild-type platelets were transplanted, tumor metastasis was reactivated [188]. Moreover, when carcinogenesis was induced in *ENPP7* (encoding alkSMase) knockout mice, a lack of alkSMase increased tumorigenesis through decreased amounts of ceramide and increased S1P and platelet-activating factor [189]. These findings suggest that SMase isoforms play differing role in cells, depending on their location within the cell and whether they are induced in cancer cells or systemically. This further elucidates the importance of cell-type-specific expression.

#### 6.2.2. Ceramide Transport

Recent research has also investigated the pathway of ceramide transport from the ER to the Golgi [65], where it is then synthesized into a complex sphingolipid. A study investigating triple-negative breast cancers demonstrated that CERT determines the signalling output of the EGFR through regulation of cellular SM levels [190]. By disrupting the endogenous activity of CERT, ceramide is unable to be exported from the ER and subsequently catabolized into SM, thus resulting in ceramide accumulation and cancer cell death. To further strengthen this conclusion, another group pharmacologically inhibited CERT, which induced ceramide-dependent apoptosis in HeLa cells [191]. Recently, a new Förster resonance energy transfer-based ceramide assay was developed to identify new CERT inhibitors [192], which provides an opportunity to develop cancer therapeutics targeting this lipid transferase.

#### 6.2.3. Ceramide Metabolism

The metabolic conversion of ceramide to its by-products is generally linked to cancer proliferation, which involves many different pathways. A gene expression analysis of CERK demonstrated that its elevated expression is associated with an increased risk of recurrence in breast cancer patients [193]. This is likely due to the rapid upregulation of CERK following chemotherapy or HER2/neu (an oncogene) downregulation, becoming essential for continued tumor survival [193]. Furthermore, the inhibition of CERK-induced apoptosis and cell cycle arrest in breast and lung cancer has been observed [194].

aCDase expression correlates with the phosphorylation of protein kinase B, or Akt, an oncogenic kinase, in human prostate tumors [195]. aCDase activates Akt through SphK1-derived generation of S1P, which leads to the stimulation of PI3K, an intracellular protein that is considered a master regulator for cancer through S1P receptor 2 [195]. A research group demonstrated the therapeutic potential of inhibiting aCDase to counteract critical growth periods in aggressive, chemo-resistant forms of prostate cancer [196]. Increased aCDase activity has also been linked to radiotherapy failure and relapse due to radiation-induced transactivation by activator protein 1 [197,198]. Inhibiting aCDase led to increased cellular radiosensitivity, which resulted in poly-ADP ribose polymerase-1 cleavage and apoptosis through a p53-dependent pathway [198]. aCDase is becoming an increasingly popular therapeutic target for various cancers to impair tumor progression and relapse [197,198,199,200,201]. For example, inhibition of aCDase blocked cell proliferation in melanomas by increasing ceramide levels and decreasing S1P levels [202], further elucidating a potential target for drug development.

nCDase is primarily expressed in the colon and small intestine, thus playing a significant role in colon carcinogenesis [203]. Its presence is required to maintain basal activity of Akt, which is responsible for the phosphorylation of GSK3B and β-catenin, resulting in the progression of tumor cells. Inhibition of nCDase results in an increase in ceramide in cells, which decreases cellular growth and increases cellular apoptosis [204]. This is explained by the activation of specific phosphatases, such as PP2A, by ceramide, which facilitates the inactivation of the oncogenic Akt pathway [205]. To support this, a recent study demonstrated that a deletion of nCDase protected mice from the onset and progression of colorectal cancer [204]. Thus, both aCDase and nCDase, through the metabolism of ceramide, affect the innate function of the Akt pathway.

Lastly for the CDases, there are three alkCDases: alkCDase1, alkCDase2, and alkCDase3. Although the involvement of alkCDase1 in cancer remains to be elucidated, it is evident that alkCDases 2 and 3 play crucial roles in this set of diseases. When targeted by p53 [206,207], *ACER2* (encoding alkCDase2) is upregulated after DNA damage to increase sphingosine levels, which induces programmed cell death through a build-up of reactive oxygen species [208]. Interestingly, a recent study reported contrasting results in hepatocellular carcinoma cells [209]. Their results showed that *ACER2* expression was associated with tumor growth and migration, possibly via SMPDL3b. These studies could suggest that the pro-survival or pro-death effect of alkCDase2 ultimately depends on the amount of its respective gene expression; it is likely that a robust overexpression of *ACER2* would result in the accumulation of sphingosine at a higher rate than S1P is able to be generated, thus having pro-apoptotic effects. Another explanation could be alkCDase2 differentially hydrolyzing ceramide species, which is seen with alkCDase3 [210]. *ACER3,* encoding alkCDase3, specifically controls the hydrolysis of unsaturated long-chain ceramides and dihydroceramides. Similarly to *ACER2*, *ACER3* regulation plays a role in hepatocellular carcinoma, as it inversely correlates with outcomes in these types of cancer patients [211]. In this study, Yin et al. (2018), knocked down *ACER3* by means of lentivirus infection, which resulted in pro-apoptotic behaviour and a reduction in tumor growth [211]. It is likely that *ACER2* and *ACER3* work together to regulate cell proliferation and cell survival. Determining the crystal structure of alkCDase3 elucidated the possibility of the structure-based discovery of small molecules to manipulate their activity for therapeutic interventions [212], providing an opportunity to modulate alkCDase3 activity to decrease pathogenesis in specific cancers.

A previous study demonstrated that S1P, produced by SphK1, is a crucial mediator of breast cancer-induced angiogenesis, lymphangiogenesis, and the promotion of metastasis [213]. It was also found to be overexpressed in various other tumors, including colon cancers [214], and was indicative of poor prognosis and decreased patient survival [215]. E2F1, a transcription factor shown to be involved in the cellular proliferation of cancer cells [216], binds to the promoter region of *SPHK1* to increase transcription [217], thus increasing sphingosine kinase and consequently S1P. Similarly, Sphk2 was also found to be highly expressed in lung cancer and correlated with lower overall survival when compared to patients that exhibited low levels of Sphk2 [218]. A recent study demonstrated that rapid phosphorylation of SphK2 by epidermal growth factor (EGF) or phorbol 12-myristate 13-acetate (PMA), a known tumor-promoting agent, resulted in an increase in kinase activity and thus enhanced EGF-induced chemotaxis of human breast cancer cells [219].

Overall, the pro-survival and pro-apoptotic functions of each sphingolipid can be manipulated through the enzymes involved in their metabolism to serve as promising therapies for a variety of cancer-types.

### 6.3. Metabolic Diseases

There has been emerging evidence on the involvement of sphingolipids, specifically ceramide and sphingosine, in obesity, insulin resistance, and fatty liver disease, all of which will be discussed in this section (summarized in Table 4).

As explored in the previous section, metabolically formed ceramide has downstream effects on Akt (encoded by *AKT1*), as well as pro-apoptotic signalling. However, in the context of insulin, Akt also stimulates the translocation of glucose transporters onto the cell membrane to increase the insulin-dependent transport of glucose into the cell [220]; thus, increased ceramide generation inhibits glucose uptake and glycogen synthesis [221]. This mechanism leads to the onset and progression of insulin resistance and subsequent type 2 diabetes. Additionally, the ceramides present in low-density lipoprotein particles have been proven to be adequate at inducing insulin resistance [222], which could help explain obesity-induced diabetes. Furthermore, CerS6, involved in C16 ceramide synthesis, is elevated in the adipose tissue of obese humans, which also correlates with insulin resistance [223]. That study then looked at mice that were deficient in CerS6 and found that they were protected from weight gain and glucose intolerance induced by a high fat diet [223]. Moreover, increased metabolism of ceramide, through an overexpression of aCDase, resulted in a reduction in hepatic steatosis in mice [224]. In addition, blocking S1P receptors and the Akt signalling pathway inhibited S1P-induced PPARy (a transcriptional regulator activated in obesity and diabetes) expression, diminishing pro-steatotic effects in mice [225]. These studies elucidate the importance of both ceramide and S1P in the pathology of metabolic disease.

Ceramide production is also provoked by inflammatory cytokines, such as interleukins and tumor necrosis factor, resulting in subsequent insulin-secreting pancreatic β-cell dysfunction and inhibition [226,227,228]. Β-cell dysfunction is also influenced by S1P, as the deletion of sphingosine phosphatase 2 caused β-cell ER stress [229]. This reveals a possible juncture in the sphingolipid signalling pathway that plays a crucial role in the development of type 1 diabetes. The alteration in sphingolipids are likely secondary effects to a mutation in *INS*, the gene encoding insulin, and responsible for triggering type 1 diabetes. The attenuation of ceramide synthesis *de novo* ameliorates glucose homeostasis, thus confirming its essential role in this pathway [230]. Diabetic complications and the lipotoxic events that precede them can also be traced back to the accumulation of ceramide in tissues [231]. Examples of these events include increased apoptosis in the kidney and eye, impaired islet cell growth and differentiation in the pancreas, decreased vascular reactivity and cardiomyopathy in the cardiovascular system, and impacts on the insulin and leptin signalling pathways in the CNS, amongst others. As a precursor to many biological functions and pathways, the manipulation of ceramide has profound effects on many cellular processes.

SM also plays a role in reducing the ability of cells to utilize glucose, and its knockout has demonstrated an improvement in whole-body insulin sensitivity and the amelioration of high-fat diet induced obesity in mice [232]. As mice aged, a reduction in white adipose tissue and lipoprotein lipase activity was also observed [233]. These results suggest that cellular ceramide may not be the only mediator of insulin resistance and obesity but is likely just a piece of the puzzle. The role of each of these sphingolipids also differed in different cell types; ceramides antagonize insulin signalling in myotubes and adipocytes, whereas fluctuations in GlcCer levels only affect adipocytes [234]. In addition, deoxysphingolipids are significantly elevated in patients with impaired fasting glucose, metabolic syndrome, and type 2 diabetes, proving to be a reliable biomarker for these conditions [235]. However, this was not seen in patients with type 1 diabetes, suggesting that glucose levels may not directly contribute to deoxysphingolipid formation [236]. Type 2, but not type 1, diabetes is typically associated with high triglycerides and has been shown to correlate with deoxysphingolipid levels [236]. Further research is required to determine how these metabolic pathways are interrelated.

In this section, we argued that ceramide is likely not the sole mediator for diabetes, and associated diseases, but an important precursor and signalling molecule that contributes to the resultant pathologies.

### 6.4. Neurodegenerative Diseases

Sphingolipids are essential for cell-to-cell signalling and neuronal membrane composition, so it is no surprise that most neurodegenerative diseases are accompanied by alterations in sphingolipid composition. Here, we re-visit the S1P:ceramide rheostat to briefly explain the role of sphingolipids in Alzheimer’s disease (AD) and Parkinson’s disease (PD) through the influence they have on neuronal growth and differentiation. The sphingolipids involved in AD and PD are summarized in Table 5.

The aggregation of amyloid plaques, extracellular plaques composed mainly of β-amyloid peptide (Aβ), are a hallmark pathology for AD. These plaques are a product of the amyloid precursor protein (APP; encoded by *APP*) that is broken down by presenilin proteins, encoded by the *PSEN1* and *PSEN2* genes. Considering that the neuronal plasma membrane is a primary target for Aβ, it is expected that sphingolipids are involved in the conformational changes occurring during their formation. Aβ interacts with SM and GalCer to alter membrane composition, as GM1 promotes Aβ fibril formation and, as a result, the formation of Aβ plaques [7,237,238]. The plaques stimulate resident immune cells and release induced nitric oxide synthase [239]. Consequently, nitric oxide induces the activation of both aSMase and nSMase in the neuronal lysosomes and cellular membranes, respectively [240,241,242]. The knockdown of both enzymes resulted in cellular resistance to Aβ-induced apoptosis, results that were anticipated due to the role of ceramide in cellular apoptosis [243]. S1P was also reduced in the brains of AD patients, potentially contributing to a larger accumulation of ceramide due to a shift in the enzymatic pathways favouring its production [244].

A similar pattern of sphingolipid accumulation is observed in alpha-synuclein (α-syn) aggregation, a marker of PD. Overexpression of α-syn (encoded by *SNCA*) leads to a disruption in the ceramide/SM recycling pathway, favouring the production of ceramide, resulting in accumulation and ultimately neurodegeneration [245]. Additionally, disruption of GlcCer leads to dysfunction of the lysosome and the stabilization of toxic α-syn with consequential neuronal cell death [246]. This suggests that GlcCer and α-syn interact; the slower GlcCer is metabolized, the more α-syn aggregates can form. A recent study supported the co-localization and interaction of GlcCer and α-syn; however, more research is necessary to determine the nature of their interaction [247]. Moreover, α-syn can inhibit the activity of lysosomal GlcCer in neurons of PD patients [248], further confirming the previous claim. Lastly, a recent study suggests that deoxysphingolipids, namely deoxydihydroceramide and deoxyceramide, could be promising therapeutic targets for both PD and AD, as they have been shown to be associated with cellular senescence [249]. However, their exact role in these neurodegenerative diseases remain to be elucidated.

AD and PD are complex neurodegenerative diseases that are a result of disruption in several pathways; however, discovering the role of sphingolipids in the progression of these diseases can drive future therapeutics research.

### 6.5. Lysosomal Storage Disorders

LSDs are a group of inherited metabolic diseases that are a result of an abnormal buildup of lipids within the cell’s lysosome, resulting in cellular toxicity. Typically, a monogenic mutation results in the cell’s inability to produce a specific hydrolytic enzyme or activator protein, thus resulting in an accumulation of the associated enzyme substrate. The accumulation of substrate leads to lysosomal swelling and, consequently, cellular apoptosis. Although the pathological lysosomal storage of sphingolipids can be seen in many cell types, the central and peripheral nervous system are particularly vulnerable to these and are affected by two-thirds of LSDs [250]. There are over 70 known LSDs, all ranging in severity and frequency. The onset of these diseases can occur during infantile, juvenile, or adult stages of life and are based on the location of the mutation and residual enzyme, or activator protein, activity. The diseases discussed in this section and the affected sphingolipids are summarized in Table 6.

#### 6.5.1. Gaucher Disease

Gaucher is the most common LSD in the population, with a prevalence of 1/40,000 [251]. There are three forms of Gaucher disease (GD): Type I (asymptomatic), Type II (acute symptoms), and Type III (severe symptoms), which are caused by a homozygous mutation in the *GBA1* gene. This disease is characterized by a deficiency of glucocerebrosidase, thus resulting in an accumulation of glycolipid GlcCer. Less commonly, GD can also be caused by a deficiency in saposin C (a mutation in *PSAP* gene), without affecting glucocerebrosidase [252]. Saposin C is a necessary activator for glucocerebrosidase [253,254] and also protects it from proteolytic degradation [255]. Current animal models for this disease focus on knocking out both glucocerebrosidase and saposin C activity in mice [256].

Patients with Type I GD and carriers of the *GBA1* mutation have been found to be predisposed to Parkinson’s disease [257,258,259,260]. As discussed in the previous section, glucosylceramide influences the formation of amyloids, which then aggregate to form Lewy bodies in nerve cells in PD [261].

#### 6.5.2. Niemann–Pick Disease

Niemann–Pick disease (NPD) is a rare disorder that is characterized by the deficiency of aSMase (types A and B; mutation in *SMPD1* gene) or the inability to traffic cholesterol and other lipids throughout the cell (type C; mutations in *NPC1* or *NPC2).*

NPD Type A and B results in an inability to catabolize SM, which leads to abnormal synapse formation and function [262,263,264]. In addition, aSMase also plays a crucial role in sphingolipid homeostasis and turnover in the lysosome.

*NPC1* encodes a large membrane glycoprotein with late endosomal localization, whereas *NPC2* encodes small soluble lysosomal proteins that bind cholesterol [265,266]. The respective proteins are located in the membrane and lumen of the late endosome, and function cooperatively for lipid transport [267,268]. Disruption in either of these result in the inability of the cells to process and utilize endocytosed cholesterol, thus increasing cholesterol storage and altering sphingolipid metabolism. In neurons, GM2 and GM3 accumulation was also observed in human tissue [269]. As the main function of cholesterol is maintaining cellular membrane integrity and fluidity, it is no surprise that sphingolipids are affected by this disease.

SM and cholesterol often co-localize to form SM-cholesterol-enriched membrane microdomains, which are essential in cell signalling [270,271]. Thus, as anticipated, Type A and B abnormalities in cholesterol metabolism are also seen [272], as well as secondary accumulation of SM in Type C (previously mentioned).

#### 6.5.3. GM1 Gangliosidoses

GM1 Gangliosidoses are caused by defective activity of β-galactosidase (encoded by *GLB1*), which is essential for GM1 ganglioside breakdown within the lysosome. GM1 is important for neuronal cell function and its accumulation leads to destruction of the nervous system. β-galactosidase cleaves the terminal β-galactose moiety of GM1, along with other GSLs; however, GM1 accumulation is the most prominent in this disease. Hydrolysis of GM1 also requires the presence of an activator protein, such as GM2A or saposin B. There are three forms of GM1 gangliosidoses: Type 1 (infantile onset), Type 2 (late infantile or juvenile onset), and Type 3 (adult onset); all of which are dependent on the location of the gene mutation. The disease severity correlates with the residual catalytic activity of the mutant enzyme [273]; the more functioning enzyme, the less severe phenotype. Patients with the infantile variant of GM1 gangliosidosis have close to 0.1% of normal catalytic activity [274], whereas late-onset patients have values closer to 10% [275].

#### 6.5.4. GM2 Gangliosidoses

This group of disorders is characterized by the excessive accumulation of GM2 within the cell’s lysosome, eventually leading to neuronal apoptosis and widespread neurodegeneration. In a properly functioning cell, the metabolism of GM2 into GM3 is mediated by the HexA enzyme, which is formed through the dimerization of two subunits, the α (encoded by *HEXA)* and β (encoded by *HEXB)*. More specifically, HexA hydrolyses the GalNAcβ residue present in GM2, as well as in GD2, GT2, and Gb4, among other GSLs [276]. The GM2A protein (encoded by *GM2A*)*,* an essential cofactor of HexA, functions to present GM2 to the HexA α-subunit. Mutations in any of the genes encoding these proteins result in GM2 gangliosidoses, which are manifested in three forms: Tay-Sachs (mutation in *HEXA*), Sandhoff (mutation in *HEXB*), and AB-Variant (mutation in *GM2A*). Although disruptions in HexA and GM2A also impact other metabolic pathways, it is GM2 that is the primary storage substrate in the lysosomes of neuronal cells in GM2 gangliosidoses patients [277]. Similar to GM1 gangliosidoses, there is an inverse correlation with the amount of residual catalytic activity and the disease severity.

## 7. Conclusions

Understanding the mechanisms behind sphingolipid structures, biosynthesis, and catabolism is essential in appreciating the fundamental role they play in small- and large-scale functions. From intra- and extracellular signalling to proliferative and pro-apoptotic effects, cell membrane and lipid composition play an integral role in the overall functioning and development of mammals. Even minor disruptions in pathways associated with sphingolipids can have a detrimental effect on cellular viability, thus resulting in the pathologies discussed in this review, among many others. Our current knowledge has continued to build a solid foundation for the development of sphingolipid therapies through the manipulation of their degradation or accumulation within the lysosome or plasma membrane. Potential therapeutics could include enzyme replacement, substrate reduction, or gene therapies to counteract the respective imbalance in homeostasis. Future research should focus on elucidating the impact sphingolipids have on other cellular pathways to continue expanding our knowledge on their interaction with other enzymes. S1P metabolism has been a promising enzyme for drug targets in clinical practice relating to brain disorders and its receptors are currently in clinical development (O’Sullivan + Dev, 2016). Several preclinical studies have also focused on reducing ceramide levels and boosting their metabolism towards the synthesis of C1P and/or S1P, using specific kinase activators [278]. A deeper understanding of these mechanisms would enhance therapeutic perspectives and offer a new route for drug development.

## Figures and Tables

**Table 1:** Examples and summary of mammalian sphingoid structure.

Classification	Sphingolipid	IUPAC Name	Structure
Sphingoid base	Sphingosine	(2S,3R)-2-aminooctadec-4-*trans*-ene-1,3-diol	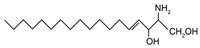
	Dihydrosphingosine (Sphinganine)	(2R,3S)-2-aminooctadecane-1,3-diol	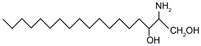
	Phytosphingosine	(2S,3S,4R)-2-aminooctadecane-1,3,4-triol	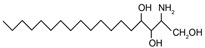
Sphingoid base derivatives	Sphingosine-1-phosphate	{[(4E)-2-amino-3-hydroxyoctadec-4-en-1-yl]oxy} phosphonic acid	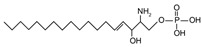
	Sphinganine-1-phosphate	{[(2S,3R)-2-amino-3-hydroxyoctadecyl]oxy}phosphonic acid	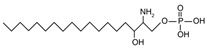
	Phytosphingosine-1-phosphate	{[(2S,3S,4R)-2-amino-3,4-dihydroxyoctadecyl]oxy}phosphonic acid	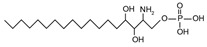
Deoxysphingolipids	1-Deoxysphinganine	[(2S,3R)-3-hydroxyoctadecan-2-yl]azanium	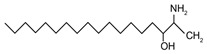
	1-Deoxysphingosine (Hypothesized structure)	2S-amino-4E-octadecen-3R-ol	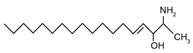
	1-Deoxysphingosine (Confirmed structure by Steiner et al., 2016)	[(2S,3R,14Z)-2-amino-14-octadecen-3-ol]	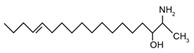
	1-Deoxymethylsphinganine	(2R)-1-aminoheptadecan-2-ol	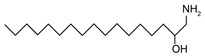
Methyl-branched sphingoid base	meC18SO (discovered by Lone et al., 2020)	16S-methyl-sphingosine	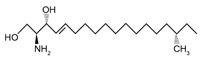

**Table 2:** Inflammatory diseases.

Disease	Protein(s) Involved	Gene(s)	Affected Sphingolipid	MIM
Cystic Fibrosis	Cystic transmembrane conductance regulator	*Cftr*	Ceramide	219700
Asthma	ORMDL3 sphingolipid biosynthesis regulator 3	*ORMDL3*	Sphingosine-1-phosphate and ceramide	600807
Irritable bowel disease	Interleukin-6	*IL6*	Sphingomyelin, sphingosine, ceramide, sphingosine-1-phosphate, ceramide-1-phosphate	612244

**Table 3:** Cancer (Note: this is not an extensive list of involved proteins, just ones that were covered in this section).

Disease	Protein(s) Involved in Ceramide Synthesis and Tumor Suppression	Protein(s) Involved in Ceramide Metabolism and Pro-Survival	MIM
Breast Cancer	CERS6, aSMase	CERT, CERK, GCS, SphK2	114480
Colorectal Cancer	SPL	-	114500
Head and neck cancer	CERS1, CERS6,	-	275355
Lung cancer	CERS6	SphK1, SphK2	211980
Leukemia	CERS1, aSMase	-	613065
Pheochromocytoma	CERS2	-	171300

CERS: ceramide synthase; aSMase: acid sphingomyelinase; CERT: ceramide transport protein; CERK: ceramide kinase; GCS: glucosylceramide synthase; SphK: sphingosine kinase; SPL: sphingosine-1-phosphate lyase.

**Table 4:** Metabolic diseases.

Disease	Protein(s) Involved	Gene(s)	Affected Sphingolipid	MIM
Diabetes type 1	Tumor necrosis factor α, interleukins	*INS*	Ceramide, sphingosine-1-phosphate	222100
Diabetes type 2	Protein kinase B (Akt)	*AKT1*	Ceramide, sphingosine-1-phosphate	125853

**Table 5:** Neurodegenerative diseases.

Disease	Protein(s) Involved	Gene(s)	Affected Sphingolipid	MIM
Alzheimer’s Disease	β-amyloid	*APP*; *PSEN1*; *PSEN2*	Sphingomyelin, galactosylceramide, GM1 ganglioside, ceramide, sphingosine-1-phosphate	104300
Parkinson’s Disease	α-synuclein	*SNCA*	ceramide, glycosylceramide	168600

**Table 6:** Lysosomal storage disorders.

Disease	Protein(s)/Enzymes	Gene(s)	Affected Sphingolipid	MIM
Gaucher Disease	Glucocerebrosidase	*GBA1*	Glucosylceramide	230800 (Type 1); 230900 (Type 2); 231000 (Type 3)
Niemann–Pick Disease (Type A and B)	Acid Sphingomyelinase	*SMPD1*	Sphingomyelin	257200 (Type A); 607616 (Type B)
Niemann–Pick Disease (Type C)	NPC1 or NPC2	*NPC1* or *NPC2*	Sphingomyelin, GM2 ganglioside, GM3 ganglioside	607623 (*NPC1*); 607625 (*NPC2*)
GM1 Gangliosidoses	β-galactosidase	*GLB1*	GM1 ganglioside	230500 (Type 1); 230600 (Type 2); 230650 (Type 3)
GM2 Gangliosidoses	Hex A (Tay-Sachs and Sandhoff) or GM2AP (AB-Variant)	*HEXA, HEXB* or *GM2A*	GM2 ganglioside	272800 (Tay-Sachs); 268800 (Sandhoff); 272750 (AB-Variant)

## Abbreviations

SMPDL3b	sphingomyelin phosphodiesterase acid-like 3b
LSDs	lysosomal storage disorders
LCB	long-chain bases
ER	endoplasmic reticulum
NADPH	nicotinamide adenine dinucleotide phosphate
FAD	flavin adenine dinucleotide
PHS	phytosphingosine
S1P	sphingosine-1-phosphate
PSLs	phosphosphingolipid
GSLs	glycosphingolipids
SM	sphingomyelin
C1P	ceramide-1-phosphate
GlcCer	glucosylceramide; glucocerebroside
GalCer	galactosylceramide; galactocerebroside
LacCer	lactosylceramide
Gal	galactose
GalNAc	N-acetylgalactosamine
Kdn	2-keto-3-deoxy-nononic acid
Neu5Ac	N-acetylneuraminic acid
Neu5Gc	N-glycolylneuraminic
CoA	coenzyme-A
SPT	serine palmitoyltransferase
ORMDL	orosomucoid-like protein
KDSR	3-ketodihydrosphingosine reductase
CerS	ceramide synthase
SMase	sphingomyelinase
CDase	ceramidase
SphK	sphingosine kinase
CERT	ceramide transport protein
SMS	sphingomyelin synthase
DAG	diacylglycerol
ERK	extracellular signal-regulated kinase
CERK	ceramide kinase
CPTP	ceramide phosphate transfer protein
UDP-Gal	uridine diphosphate galactose
UGCG	UDP-glucose ceramide glucosyltransferase
BMP	bis(monoacylglycerol)phosphate
aSMase	acid sphingomyelinase
L-aSMase	lysosomal acid sphingomyelinase
S-aSMase	secretory acid sphingomyelinase
nSMase	neutral sphingomyelinase
alkSMase	alkaline sphingomyelinase
NPP	nucleotide pyrophosphatase/phosphodiesterase
LLBP	lysosomal lipid binding proteins
GM2A	GM2 activator protein
GH	glycosyl hydrolase
HexA	β-hexosaminidase A
aCDase	acid ceramidase
nCDase	neutral ceramidase
alkCDase	alkaline ceramidase
LPP1-3	lipid phosphate phosphatases
SPL	sphingosine-1-phosphate lyase
PLP	pyridoxal 5′-phosphate
GPCR	G-protein coupled receptor proteins
CT	cholera toxin
SV40	simian virus 40
JNK	c-Jun N-terminal kinases
BMDM	primary bone-marrow-derived macrophages
mTOR	mammalian target of rapamycin
GFR	growth factor receptors
EGFR	epidermal GFR
ERM	ezrin, radixin, and moesin
CF	cystic fibrosis
COPD	chronic obstructive pulmonary disease
SHRSP	spontaneously hypertensive stroke-prone
RTK	receptor tyrosine kinases
LT	leukotriene
5-LO	5-lipoxygenase
AA	arachidonic acid
PLA2	phospholipase A2 activity
IBD	inflammatory bowel disease
HDAC1	histone deacetylase 1
PMA	horbol 12-myristate 13-acetate
EGF	epidermal growth factor
AD	Alzheimer’s disease
PD	Parkinson’s disease
Aβ	β-amyloid peptide
APP	amyloid precursor protein
α-syn	alpha-synuclein
GD	Gaucher disease
NPD	Niemann–Pick disease
